# Liraglutide Alleviates Hepatic Steatosis and Liver Injury in T2MD Rats via a GLP-1R Dependent AMPK Pathway

**DOI:** 10.3389/fphar.2020.600175

**Published:** 2021-03-04

**Authors:** Rui Zhou, Chuman Lin, Yanzhen Cheng, Xiaoyun Zhuo, Qinghua Li, Wen Xu, Liang Zhao, Li Yang

**Affiliations:** ^1^ Department of Nutrition, Zhujiang Hospital, Southern Medical University, Guangzhou, China; ^2^ Department of Endocrinology, Zhujiang Hospital, Southern Medical University, Guangzhou, China; ^3^ Department of Pathology, Nanfang Hospital, Southern Medical University, Guangzhou, China; ^4^ Department of Pathology, School of Basic Medical Sciences, Southern Medical University, Guangzhou, China; ^5^ Department of Endocrinology and Metabolism, Guangdong Provincial Key Laboratory of Diabetology, Third Affiliated Hospital of Sun Yat-sen University, Guangzhou, China

**Keywords:** liraglutide, hepatic steatosis, liver injury, T2DM, PPARα

## Abstract

Non-alcoholic fatty liver disease (NAFLD), ranging from non-alcoholic fatty liver to non-alcoholic steatohepatitis, can be prevalent in patients with type 2 diabetes mellitus (T2DM). However, no antidiabetic drug has been approved for the treatment of NAFLD in T2DM patients. Multiple daily injections of basal-bolus insulin are often the final therapeutic option for T2DM. We found that insulin treatment aggravated hepatic steatosis and oxidative stress in Zucker diabetic fatty (ZDF) rats. In addition to glycaemic control, we demonstrated the stimulatory role of liraglutide in relieving hepatic steatosis and liver injury in ZDF rats. Interestingly, liraglutide could also alleviate insulin-aggravated hepatic fatty accumulation. The glucagon-like peptide-1 (GLP-1) agonists liraglutide and Ex-4 activated the expression of peroxisome proliferator-activated receptor alpha (PPARα) via a GLP-1 receptor-dependent 5′ AMP-activated protein kinase pathway. As a nuclear transcription factor, PPARα could mediate the effect of GLP-1 in alleviating hepatic steatosis by differentially regulating the expression of its target genes, including acetyl CoA carboxylase and carnitine palmitoyl transferase la both *in vitro* and *in vivo*. Moreover, GLP-1 could relieve liver injury by decreasing oxidative stress stimulated by hepatic steatosis. Insulin might aggravate hepatic steatosis and liver injury by inhibiting GLP-1R expression. The findings indicate the feasibility of liraglutide treatment combined with basal insulin in attenuating hepatic steatosis and liver injury in ZDF rats. This knowledge, and the evidence for the underlying mechanism, provide a theoretical basis for the combination treatment recommended by the latest clinical practice guidelines for T2DM.

## Introduction

Diabetes mellitus (DM) is a common non-communicable disease that affects the global population, including an estimated 11.6% of the Chinese adult population (Wang et al., 2017). Approximately 50–70% of diabetics in China have type 2 DM (T2DM). They are more likely to develop non-alcoholic fatty liver disease (NAFLD) (Fruci et al., 2013). NAFLD is strongly related to metabolic risk factors such as DM, obesity and dyslipidaemia (Cazzo et al., 2018). Diabetes increases the mortality rate of hepatic diseases by accelerating the progression of NAFLD from excessive hepatic fat deposition into non-alcoholic steatohepatitis (NASH), hepatic fibrosis and even hepatic carcinoma (Diehl and Day, 2017).

T2DM is characterized by insulin resistance and lesions of pancreatic beta cells, resulting in a worsening glycaemic control and an increasing use of antidiabetic drugs including insulin (Kahn et al., 2009). If synthetic antidiabetic drugs or basal insulin is unable to achieve glycaemic control, the final therapeutic step is often multiple daily injections of basal-bolus insulin (American Diabetes Association, 2018). However, in most cases, it is still difficult to achieve excellent clinical glycaemic control due to the high risks of hypoglycaemia and weight gain caused by inappropriate insulin dosage. Relief of NAFLD in patients with T2DM is also difficult (Horie et al., 2018). A combination treatment has been suggested by the latest American Association of Clinical Endocrinology/American College of Endocrinology (AACE/ACE) clinical practice guidelines (American Diabetes Association, 2019; Garber et al., 2019).

Glucagon-like peptide-1 (GLP-1) is an incretin hormone that is secreted from intestinal L-cells and circulates through the ingestion of nutrients (Baggio and Drucker, 2007). The pleiotropic functions of GLP-1 in mammals include promoting insulin secretion, suppressing glucagon release, slowing gastric emptying, and minimizing insulin-mediated glucose uptake (Guo et al., 2017). As a new class of antidiabetic drugs, GLP-1 agonists like exenatide and liraglutide are already in use for the clinical treatment of T2DM. In addition to improved glycaemic control, GLP-1 also effectively reduces lipid load and free fatty acid (FFA)-induced liver steatosis (Gupta et al., 2010; Wajcberg and Amarah, 2010; Cuthbertson et al., 2012). The combination treatment with GLP-1 agonists and basal insulin synergistically lowers the level of glucose and reduces postprandial glycaemic excursion (Balena et al., 2013; Campbell and Drucker, 2013; Holst and Vilsboll, 2013). This combination also allows a reduced insulin dosage, lessens the risk of insulin-induced hypoglycaemia, and reduces weight gain. The combination treatment with liraglutide and metformin also was reported to dramatically reduce the body weight and intrahepatic lipid of T2DM patients with NAFLD (Yan et al., 2018). Unfortunately, most of the conclusions came from clinical trials, and the underlying mechanisms remain elusive.

Peroxisome proliferator-activated receptor alpha (PPARα) is a ligand-activated transcription factor of the NR1C nuclear receptor subfamily (Pawlak et al., 2015). In rodents, high PPARα expression has been detected in tissues characterized by a high rate of fatty acid oxidation, including brown adipose tissue, liver, kidney and heart (Escher et al., 2001; Bookout et al., 2006). Hepatic PPARα deletion in mouse models results in impaired fatty acid catabolism and hepatic lipid deposition (Montagner et al., 2016). Several studies have indicated that PPARα is a crucial regulator of lipid metabolism in the liver through the activation of target genes (Kersten et al., 1999; Leone et al., 1999; Hashimoto et al., 2000; Pawlak et al., 2015). In patients with atherogenic dyslipidaemia, synthetic PPARα agonists (fibrates) can lower the levels of plasma triglycerides (TGs) and low-density lipoprotein (LDL) while raising the levels of high-density lipoprotein cholesterol (HDL-C) (Staels et al., 2008).

GLP-1 receptor (GLP-1R) is present on human hepatocytes (Gupta et al., 2010). Thus, GLP-1 might reduce NAFLD in T2DM patients by directly acting on hepatic GLP-1Rs. As well, since PPARα is also highly expressed in human hepatocytes (Kersten and Stienstra, 2017), it is reasonable to hypothesize that PPARα is involved in GLP-1 mediated lipid metabolism in the liver.

In this study, Zucker diabetic fatty (ZDF) mice were treated with the combination of liraglutide and insulin. The effects on hepatic steatosis and liver injury were studied. The involvement of PPARα in GLP-1 mediated amelioration of hepatic steatosis, oxidative stress and liver injury was investigated *in vivo* and *in vitro*. The study explored the feasibility of the liraglutide–insulin combination treatment to alleviating T2DM-induced or insulin aggravated NAFLD and provide the first details of the mechanism of these effects. The findings will inform improvements to clinical practice guidelines for T2DM.

## Materials and Methods

### Animal Studies

Male ZDF rats (Fa/Fa, *n* = 30) and control lean rats (+/Fa, *n* = 7) were obtained from the Laboratory Animal Center of Vital River (Beijing, China; license number, SYXK (Yue) 2011-0074). The average weight of ZDF and lean rats at 8 weeks of age was 286 and 233 g, respectively. Rats were housed in an identical room with free access to a high-fat diet and purified water throughout the experiment. A high-fat diet (Purina 5008, PA) was provided from 8 weeks of age to the end of the study. After four weeks of induction, the glucose levels of the rats ranged between 19.3.3 and 33.3 mmol/L (average 26.53 ± 0.69 mmol/L). At of 12 weeks age, the ZDF rats were randomly divided into four groups: 1) diabetic control group (PC, *n* = 7); 2) diabetic group treated with insulin glargine (INS, *n* = 7); 3) diabetic group treated with the saxagliptin inhibitor dipeptidyl peptidase 4 (DPP-4) (Saxag, *n* = 7); and 4) diabetic group treated with the GLP-1 analogue liraglutide (Lirag, *n* = 9). The normal control group (NC, *n* = 7) and PC group were treated with 0.9% normal saline. Insulin glargine was intravenously injected into the INS group once a day and the dosage was set according to the changes in glucose levels of the Lirag group throughout the experiment. The Saxag group received saxagliptin (AstraZeneca, London, England) by intragastric gavage at a dosage of 5 mg/kg/24 h. The Lirag group was administered liraglutide (Novo Nordisk, Copenhagen, Denmark) at a dose of 200 μg/kg/12 h for 8 weeks, as previously described (Hayes et al., 2011). Glucose levels, body weight and food intake were measured every week. At the age of 20 weeks, the rats were anesthetized with 2.5% pentobarbital sodium and the left ventricular was punctured for blood sampling. The livers of rats were fixed in 4% paraformaldehyde or frozen in liquid nitrogen. All animal experiments were approved by the Institutional Animal Care and Use Committee of the Southern Medical University (Guangzhou, China).

### Cell Culture and Treatment

Normal hepatocytes LO2 and hepatoma HepG2 cells were obtained from the Cell Bank of the Chinese Academy of Sciences (Shanghai, China). Exendin-4 was purchased from MedChemExpress (United States). Exendin Fragment 9-39 (Exendin-9) was obtained from Sigma-Aldrich (United States). All the cells were cultured in DMEM (Invitrogen; Invitrogen; Paisley, United Kingdom) with 10% fetal bovine serum (FBS; Gibco-BRL, Invitrogen; Paisley, United Kingdom) in a 5% CO2 incubator at 37°C. ShPPARα (targeting 5′-GAC​TCA​AGC​TGG​TGT​ATG​A-3′) was purchased from Sangon Biotech (Shanghai, China). Exponential growth phase cells were transfected with 2.5 μg shPPARα in reduced serum medium (OPTI-MEM-I) according to the manufacturer’s protocol at 50–70% confluence. To build the hepatocyte steatosis model, the cells were exposed to sodium palmitate (PA) at a final concentration of 0.3 μM. The cells were divided into the following groups: 1) normal control group (NC): the cells were cultured in DMEM for 24 h; 2) PA group: the cells were exposed to PA (0.3 μM) in DMEM for 24 h; 3) PA + Exendin-4 group (PA + Ex-4): the cells were co-treated with PA (0.3 μM) and Exendin-4 (100 nM) in DMEM for 24 h; 4) PA + Exendin-4+Exendin-9 group (PA + Ex-4+Ex-9): the cells were cultured in PA (0.3 μM) and Exendin-9 (100 nM) for 1 h and were then co-administered with Exendin-4 (100 nM) for 24 h. 5) Insulin group (Insulin): the cells were exposed to Insulin (100 nM) in DMEM for 24 h; 6) PA + Insulin group (PA + Insulin): the cells were co-treated with PA (0.3 μM) and Insulin (100 nM) in DMEM for 24 h; 7) PA + Exendin-4+Insulin group (PA + Ex-4+Insulin): the cells were co-treated with PA (0.3 μM), Exendin-4 (100 nM) and Insulin (100 nM) in DMEM for 24 h; 8) sh PPARα group: the cells transfected with shRNA duplexes targeting PPARα were cultured in DMEM for 48 h; 9) PA + Exendin-4+shPPARα group (PA + Ex-4+shPPARα): the cells transfected with shRNA duplexes targeting PPARα were cultured in DMEM for 48 h and were then co-administered with PA (0.3 μM) and Exendin-4 (100 nM) for 24 h.

### Blood Biochemical Analysis

After overnight fasting, blood samples were collected from left ventricular puncturing and subsequently centrifuged at 3,000 rpm for 10 min after standing at room temperature for 3 h. Total cholesterol (mM), triglycerides (mM), HDL cholesterol (mM), LDL cholesterol (mM), AST (IU/L) and ALT (IU/L) were determined by an automated biochemistry analyzer (Cobas Integra 400 Plus; Roche Diagnostics, Basel, Switzerland).

### Oral Glucose Tolerance Test

Oral glucose tolerance test was conducted on rats at the age of 20 weeks after an overnight fasting. After testing fasting blood glucose (FBG), rats were intragastrically given glucose solution at 2 g/kg body weight. The blood samples were collected from the rats’ tail vein and the plasma glucose was measured by a glucose meter 0, 30, 60, and 120 min after glucose administration. The area under the curve of glucose was calculated using the trapezoidal method (AUC = 1/4 fasting glucose +1/2 30 min glucose +3/4 60 min glucose +1/2 120 min glucose).

### Histological and Immunohistochemistry Analysis

Liver tissues were fixed in 4% paraformaldehyde for 24 h and maintained in 70% alcohol for subsequent paraffin embedding. Paraffin sections were cut at a thickness of 3 μm and hematoxylin-eosin (HE) staining was performed after removal of paraffin. The hepatic steatosis of each section was observed by optical microscope using a model BX53 instrument (Olympus, Tokyo, Japan). IHC was performed to investigate the expression of proteins in human hepatic tissues. The sections were incubated overnight with 1:100 dilutions of primary antibodies against PPARα (bs-3614R; Bioss Antibodies, Boston, MA, United States) and GLP-1R (bs-1559R; Bioss) overnight at 4°C. This was followed by incubation with goat anti-rabbit secondary antibody conjugated with 3,3′-diaminobenzidine (DAB) chromogen. Samples were examined by optical microscopy. The slides were reviewed concerning low or high expression in a blinded manner by two pathologists.

### Oil Red O Staining

LO2 cells were stained with Oil red O (ORO, Solarbio, Beijing, China) solution to visualize the accumulation of intracellular lipids. In brief, LO2 cells were washed with PBS for three times before fixing with 10% formalin for 20 min. After washing with 60% isopropanol for 5 min, the cells were stained with ORO solution for 20 min at room temperature. Then, the cells were washed by distilled water to remove the excess dyestuff and counterstained with hematoxylin. The red-stained lipid droplets were observed and photographed using a light microscope.

### RNA Isolation, Reverse Transcription and Real-Time Quantitative PCR

Total RNA was extracted using Trizol reagent (Invitrogen Life Technologies, Carlsbad, CA, United States) according to the manufacturer’s instructions. The total RNA was transcribed into cDNA using PrimeScript RT reagent Kit with gDNA Eraser (DRR047A, TaKaRa). Real-time quantitative PCR (qPCR) was carried out using SYBR Green PCR master mix (Applied Biosystems; Foster City, CA) on ABI 7500HT system. GAPDH was chosen as endogenous control. All the primers shown in Table 1 were synthesized from Sangon Biotech (Shanghai, China). The expression level of each targeted gene was normalized as fold change compared to control or reference group. Fold changes were calculated through relative quantification (2^−ΔΔCT^).

**TABLE 1:** The primers sequences used for real-time quantitive PCR.

Genes	Forward primer	Reverse primer
PPARα^H^	ATG​GTG​GAC​ACG​GAA​AGC​C	CGA​TGG​ATT​GCG​AAA​TCT​CTT​GG
PPARα^M^	AAG​GGC​TTC​TTT​CGG​CGA​AC	TGA​CCT​TGT​TCA​TGT​TGA​AGT​TCT​TCA
CPT1a^H^	CCT​CCA​GTT​GGC​TTA​TCG​TG	TTC​TTC​GTC​TGG​CTG​GAC​AT
CPT1a^M^	TTGGGCCGGTTGCTGAT	GTC​TCA​GGG​CTA​GAG​AAC​TTG​GAA
ACC^H and m^	ATG​TCT​GGC​TTG​CAC​CTA​GTA	CCC​CAA​AGC​GAG​TAA​CAA​ATT​CT
GAPDH^H^	AAG​GTC​GGA​GTC​AAC​GGA​TTT​G	CCA​TGG​GTG​GAA​TCA​TAT​TGG​AA
GAPDH^M^	GAA​CAT​CAT​CCC​TGC​ATC​CA	CCA​GTG​AGC​TTC​CCG​TTC​A

^H^, Human; ^M^, Mouse.

### Western-Blot Analysis

Western blot analysis was conducted to investigate the expression of proteins and their phosphorylation level in hepatic tissues and cells. Hepatic tissues were ground into powders with liquid nitrogen and hepatic cells were homogenized in RIPA lysis buffer (1% leagene PMSF, 1% protease and Phosphatase Inhibitor Cocktail). Proteins extracted from each group were separated by SDS-PAGE and transferred to PVDF membranes. Nonspecific binding was blocked using a blocking reagent (0.1% Tween 20 and 5% Bovine Serum Albumin in TBS) and the membranes were then incubated with specific primary antibodies overnight at 4°C. Primary antibodies (1:1000) used in this manuscript are: anti-AMPK alpha 1 and anti-p-AMPK alpha 2 (S345) obtained from Biohua (Hangzhou, China); anti-GAPDH purchased from Santa Cruz (Biotechnology, Santa Cruz, CA, United States); anti-PPARα obtained from Proteintech (Chicago, CA, United States), anti-ACC was obtained from Abcam (Cambridge, England); anti-GLP-1R (Beijing, China, Bioss), and anti-p-ACC purchased from Affinity Biosciences (OH, United States). After incubating the membrane with the appropriate secondary antibodies for 1 h at room temperature, Perkinelmer western lightning Plus-ECL was used to detect the specific bands. The immunoblots were quantified by densitometric analysis using Quantity One software (Bio-Rad, Hercules, United States).

### Reactive Oxygen Species Assessment

Intracellular accumulation of ROS was measured using a Reactive Oxygen Species assay kit (Beyotime Institute of Biotechnology) containing fluorescent probe, 2′,7′-dichloro-dihydro-fluorescein diacetate (DCFH-DA). In brief, cells were seeded in serum-free medium containing 10 μM DCFH-DA (v/v, 1:1,000) and incubated at 37°C for 30 min. The residual DCFH-DA solution was removed and cells were washed with serum-free medium three times for 5 min, and then washing with PBS. Cells were observed under a fluorescence microscope (Olympus Corporation). Moreover, intracellular fluorescence intensity were analyzed by flow cytometry (FACSCalibur, Becton–Dickinson, United States) in PBS dissolved 10,000 cells of each sample. The total intracellular ROS level is calculated as the percentage of control cells and is directly proportional to the fluorescence intensity.

### Immunofluorescence

Immunofluorescence staining was performed according to the standard protocol described previously (Liao et al., 2017). Polyclonal rabbit primary antibody to GLP-1R (Bioss, bs-1559R; 1:100), HRP-conjugated secondary antibody and DAB staining kit (CWBIO, Beijing, China) were used in the experiment. Images were captured with a laser confocal microscope (FV10-ASW, Olympus, Tokyo, Japan).

### Apoptosis Assay and TUNEL Assay

Propidium iodide (PI) and Annexin V-FITC-flow cytometry assay (BD Pharmingen, CA) was used to detect the apoptosis in the cells. Cells were stained with FITC-conjugated annexin V and PI (KeyGEN BioTECH, China, KGA107) according to the manufacturer’s instructions. After staining, the cells were analyzed by flow cytometry (FACS Calibar; Becton-Dickinson) using Cell Quest software.

TUNEL assay was processed using the *in situ* cell death detection kit (KeyGEN BioTECH, China, KGA7071). Hepatic tissues were stained according to the manufacturer’s instructions. The apoptosis index was captured using a fluorescence microscope (Olympus IX73, Japan).

### Statistical Analyses

Data were analyzed using SPSS version19.0 (SPSS, Chicago, IL, United States). The Student’s t-test and one-way ANOVA test were carried out for RT-PCR. Statistical significance was set at *p* < 0.05.

## Results

### Liraglutide Improves Glycaemic Control of Zucker Diabetic Fatty Rats

**FIGURE 1:**
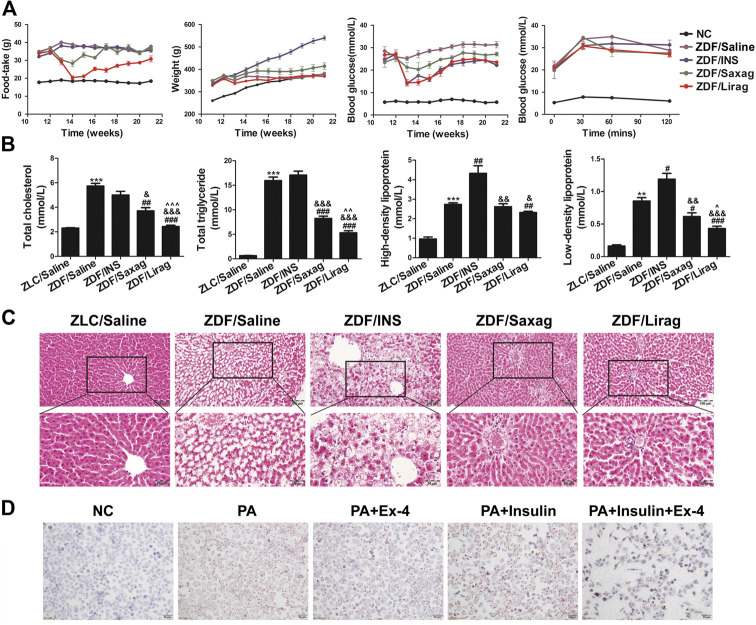
Liraglutide improves the glycemic control and alleviates hepatic steatosis of ZDF rats. **(A)** Foodtake, body weight, blood glucose and glucose tolerate of lean and indicated ZDF rats. **(B)** Serum lipid profiles of lean and indicated ZDF rats (^*^ *p* < 0.05, ^**^ *p* < 0.01, ^***^ *p* < 0.001 vs. ZLC group; ^#^ *p* < 0.05, ^##^ *p* < 0.01, ^###^ *p* < 0.001 vs. ZDF/saline group; ^&^ *p* < 0.05, ^&&^ *p* < 0.01, ^&&&^ *p* < 0.001 vs. ZDF/INS group; ^^^ *p* < 0.05, ^^^^ *p* < 0.01, ^^^^^ *p* < 0.001 vs. ZDF/Lirag group). **(C)** HE staining of hepatic tissues of lean and indicated ZDF rats. **(D)** Oil O staining indicates the lipid accumulation in indicated hepatic cells.

To confirm the effects of GLP-1 on glycaemic control, both liraglutide and saxagliptin (dipeptidyl peptidase 4 inhibitor, which can increase the secretion of endogenous GLP-1), were used to treat ZDF rats. The food intake of both compounds resulted in decreased food intake at most time points, especially 4 weeks after antidiabetic drug treatment (Figure 1A, first panel). At the end of the treatment, the cumulative food intake of ZDF rats treated with liraglutide and saxagliptin had increased to a level similar to that of the positive control group. However, the food intake of the liraglutide group remained lower than that of the other groups.

The body weights of ZDF rats were higher than those of lean rats at all time points. Insulin significantly and continuously produced an increased weight gain of the ZDF rats. Notably, liraglutide decreased the weight of ZDF rats, especially after 9 weeks of treatment (Figure 1A, second panel). However, a similar decrease in body weight was not detected in ZDF rats treated with saxagliptin.

As expected, blood glucose levels increased significantly in ZDF rats compared with lean rats. Insulin, liraglutide and saxagliptin dramatically alleviated the blood glucose level in ZDF rats (Figure 1A, third panel). The oral glucose tolerance test analysis showed that lean rats were tolerant to glucose, whereas ZDF rats had an impaired tolerance to glucose (Figure 1D). Although the fasting glucose levels in all ZDF rats subjected to different treatments were the same, liraglutide-treated rats showed a significant decrease the glucose level at each time point after oral glucose intake (Figure 1A, fourth panel). Saxagliptin also improved glucose tolerance in ZDF rats.

### Liraglutide Alleviates Hepatic Steatosis *in vivo* and *in vitro*

We also examined serum lipid profiles that included total cholesterol (TC), TG, LDL-cholesterol (LDL-C), and HDL-C in ZDF and lean rats, including. Administration of liraglutide and saxagliptin decreased the levels of TC, TG, and LDL-C in ZDF rats. The effects of liraglutide were more dramatic than those of saxagliptin (Figure 1B). Both liraglutide and saxagliptin slightly decreased HDL-C levels (Figure 1B, third panel). Conversely, insulin dramatically increased the levels of LDL-C and HDL-C in ZDF rats. The livers of ZDF and lean rats were dissected and observed by optical microscopy after HE staining. Compared with lean rats, ZDF rats exhibited severe hepatic steatosis. To our surprise, big and confluent regions of lipid were detected in the liver of insulin treated ZDF mice. In these mice, hepatic steatosis was more severe compared to control ZDF rats. Administration of liraglutide and saxagliptin completely prevented the establishment of hepatic steatosis (Figure 1C). The GLP-agonist Ex-4 significantly alleviated PA induced lipid accumulation in LO2 cells (Figure 1D). Similar to *in vivo* experiments, insulin aggravated PA induced lipid accumulation, while Ex-4 dramatically relieved the insulin aggravated lipid accumulation.

### Liraglutide Alleviates Hepatic Injury by Reducing Oxidative Stress

**FIGURE 2:**
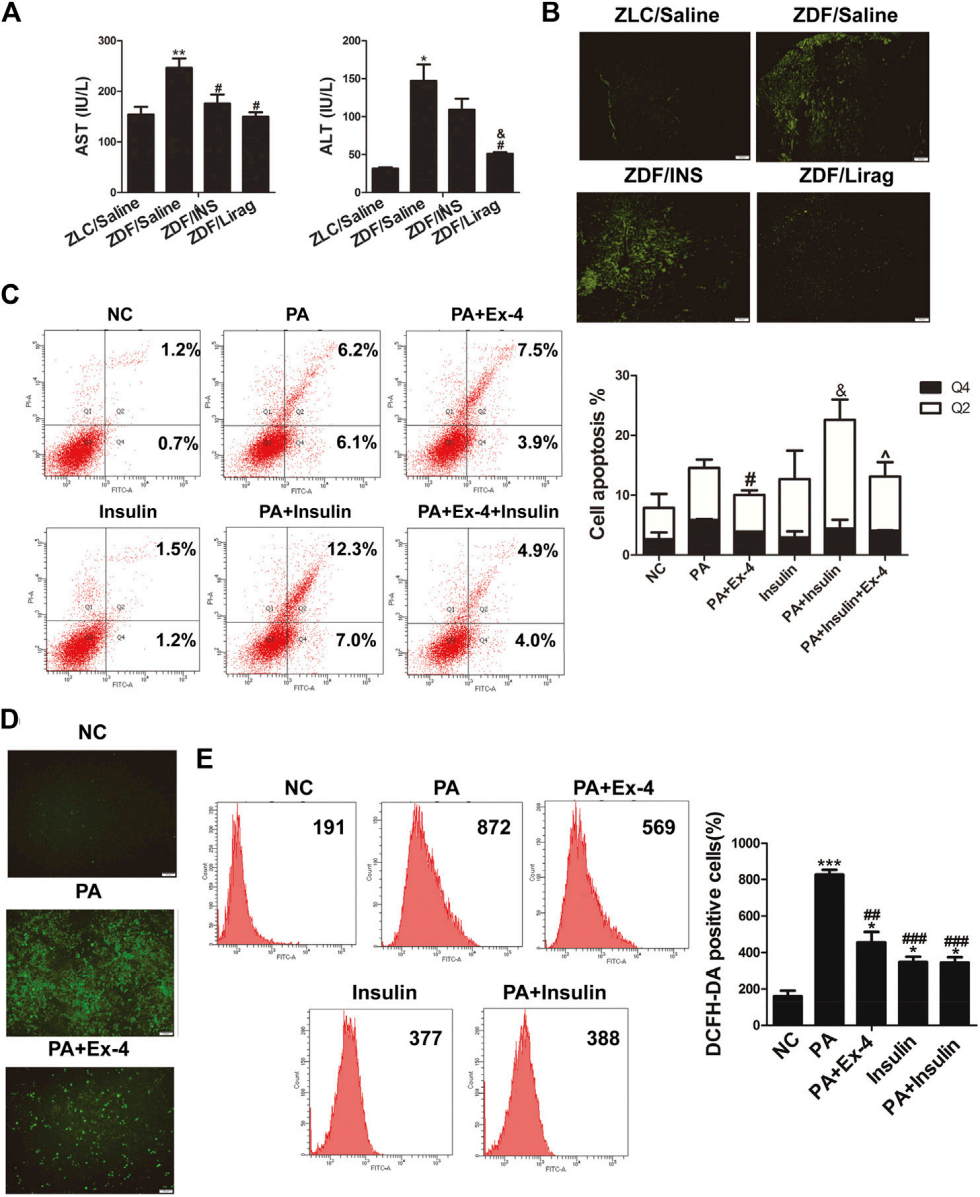
Liraglutide alleviates hepatic steatosis through increasing oxidative stress. **(A)** AST and ALT levels in the serum of lean and indicated ZDF rats (^*^ *p* < 0.05, ^**^ *p* < 0.01, ^***^ *p* < 0.001 vs. ZLC group; ^#^ *p* < 0.05, ^##^ *p* < 0.01, ^###^ *p* < 0.001 vs. ZDF/saline group; ^&^ *p* < 0.05, ^&&^ *p* < 0.01, ^&&&^ *p* < 0.001 vs. ZDF/INS group; ^^^ *p* < 0.05, ^^^^ *p* < 0.01, ^^^^^ *p* < 0.001 vs. ZDF/Lirag group). **(B)** TUNEL assay shows the liver injury of lean and indicated ZDF rats. **(C)** Annexin V-FITC/PI flow cytometry assay shows the effects of Ex-4 on PA induced apoptosis. Bars on the right panel represent percentage of cells in Q2+Q4 (^*^ *p* < 0.05, ^**^ *p* < 0.01, ^***^ *p* < 0.001 vs. NC group; ^#^ *p* < 0.05, ^##^ *p* < 0.01, ^###^ *p* < 0.001 vs. PA group; ^&^ *p* < 0.05, ^&&^ *p* < 0.01, ^&&&^ *p* < 0.001 vs. PA + Ex-4 group; ^^^ *p* < 0.05, ^^^^ *p* < 0.01, ^^^^^ *p* < 0.001 vs. shPPARα group). **(D)** DCFH-DA signals indicate the ROS level in indicated hepatic cells. **(E)** Flow cytometry shows DCFH-DA positive cells in indicated PA treated hepatic cells. Bars on the right panel represent DCFH-DA positive cells (^*^ *p* < 0.05, ^**^ *p* < 0.01, ^***^ *p* < 0.001 vs. NC group; ^#^ *p* < 0.05, ^##^ *p* < 0.01, ^###^ *p* < 0.001 vs. PA group).

The levels of aspartate aminotransferase (AST) and alanine aminotransferase (ALT) levels were detected in the blood serum of ZDF and lean rats to study liver injury. Liraglutide treatment produced decreased levels of AST and ALT levels in ZDF rats, while only AST levels were significantly downregulated in the insulin treated group (Figure 2A). The terminal deoxynucleotidyl transferase dUTP nick end labeling (TUNEL) assay was also performed on hepatic tissues of ZDF and lean rats. Compared with lean rats, the apoptosis of liver cells was dramatically increased in ZDF rats. Administration of liraglutide completely abolished the apoptosis of liver cells in ZDF rats (Figure 2B). Flow cytometry assays indicated that incubation in the presence of PA dramatically increased the apoptosis of LO2 cells, while Ex-4 significantly alleviated PA induced apoptosis (Figure 2C). Although insulin did not influence the apoptosis of liver cells, it aggravated PA induced hepatic cell apoptosis.

Increased oxidative stress has been identified as a major cause of liver injury (Lirussi et al., 2007). A reactive oxygen species (ROS) assay kit was used to detect the ROS level in LO2 cells with different treatments. PA treated hepatic cells displayed more intense DCFH-DA signals compared with the control group when examined by fluorescence microscopy. Liraglutide dramatically reduced the DCFH-DA signals in the PA treated hepatic cells (Figure 2D). Flow cytometry indicated that Ex-4 remarkably reduced the number of DCFH-DA-positive cells in PA treated hepatic cells. Insulin dramatically increased oxidative stress (Figure 2E).

### PPARα Mediates GLP-1 Induced Lipid Metabolism *In Vivo* and *In Vitro*

**FIGURE 3:**
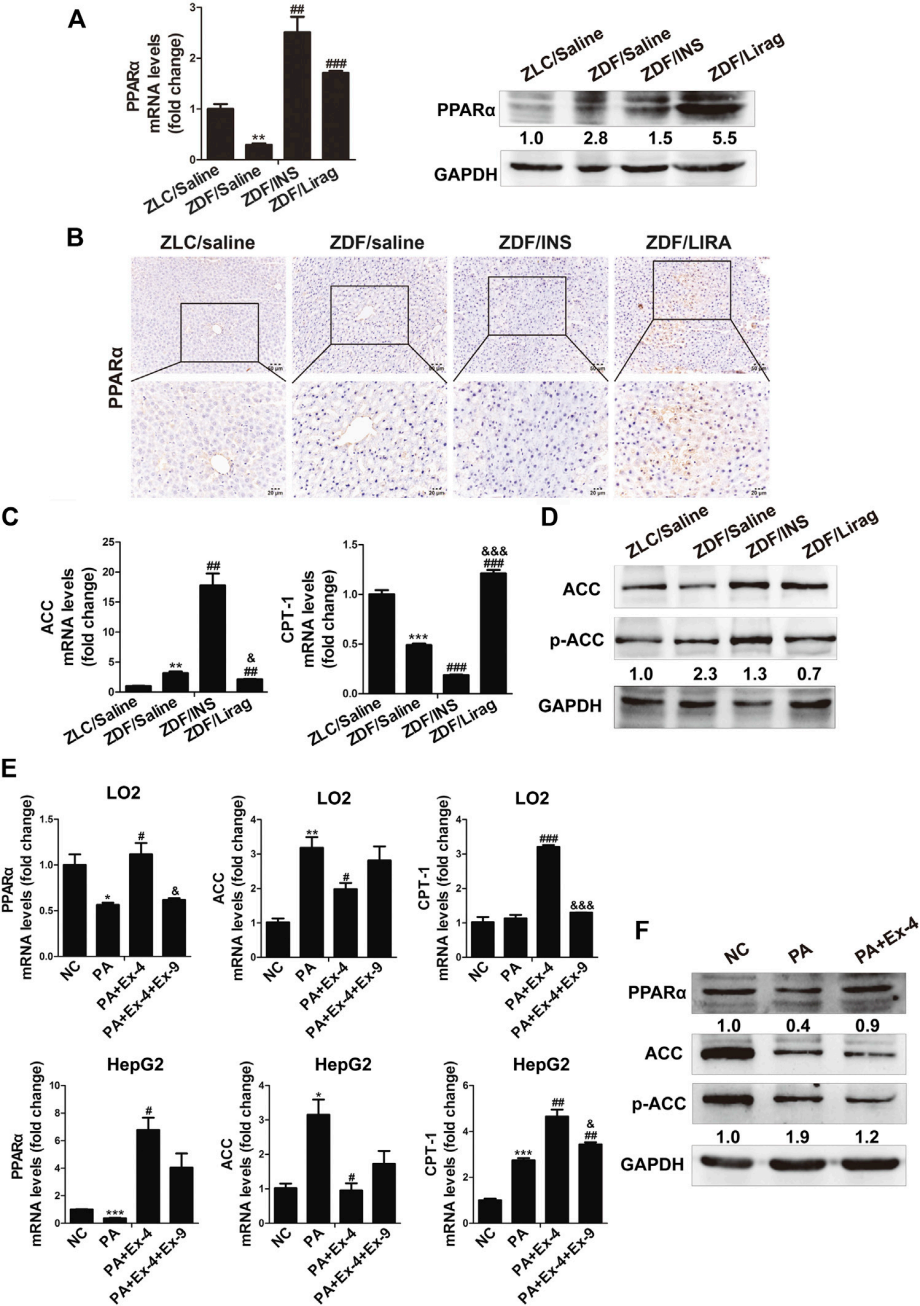
PPARα mediates GLP-1 induced lipid metabolism both *in vivo* and *in vitro*. **(A)** Real-time PCR and western blot detects the expression of PPARα in the hepatic tissues of lean and indicated ZDF rats (^*^ *p* < 0.05, ^**^ *p* < 0.01, ^***^ *p* < 0.001 vs. ZLC group; ^#^ *p* < 0.05, ^##^ *p* < 0.01, ^###^ *p* < 0.001 vs. ZDF/saline group). **(B)** IHC staining shows the expression of PPARα in the hepatic tissues of lean and indicated ZDF rats. **(C)** Real-time PCR shows the expression of ACC and CPT-1 in the hepatic tissues of lean and indicated ZDF rats (^*^ *p* < 0.05, ^**^ *p* < 0.01, ^***^ *p* < 0.001 vs. ZLC group; ^#^ *p* < 0.05, ^##^ *p* < 0.01, ^###^ *p* < 0.001 vs. ZDF/saline group; ^&^ *p* < 0.05, ^&&^ *p* < 0.01, ^&&&^ *p* < 0.001 vs. ZDF/INS). **(D)** Western blot shows the expression of ACC and p-ACC in the hepatic tissues of lean and indicated ZDF rats. **(E)** Real-time PCR and western blot shows the expression of ACC and CPT-1 in LO2 and HepG2 cells (^*^ *p* < 0.05, ^**^ *p* < 0.01, ^***^ *p* < 0.001 vs. NC group; ^#^ *p* < 0.05, ^##^ *p* < 0.01, ^###^ *p* < 0.001 vs. PA group; ^&^ *p* < 0.05, ^&&^ *p* < 0.01, ^&&&^ *p* < 0.001 vs. PA + Ex-4 group).

PPARα is a nuclear transcription factor that can activate the expression of enzymes related to fat metabolism (Pawlak et al., 2015). To confirm the involvement of PPARα in GLP-1 induced metabolism, the expression of PPARα and its target genes were detected in ZDF and lean rats. The RNA expression of PPARα was dramatically decreased in the liver of ZDF rats compared with that in lean rats. Although the expression of PPARα was increased in the liver of insulin- and liraglutide-treated ZDF rats at the transcriptional level, PPARα protein was only overexpressed in the liraglutide group (Figure 3A). This was confirmed by IHC data. Compared with lean rats, stronger PPARα staining was detected in liver sections of liraglutide-treated ZDF rats (Figure 3B). The expression of acetyl CoA carboxylase (ACC) and carnitine palmitoyl transferase la (CPT1a), two PPARα targeting genes related to fat metabolism, were studied in both hepatic tissues and cell lines. The liraglutide group showed decreased hepatic expression of ACC and p-ACC, but increased expression of CPT1a. In contrast, insulin stimulated the hepatic expression of ACC and p-ACC, but inhibited that of CPT1a (Figure 3C,D). In PA treated LO2 and HepG2 cells, administration of Ex-4 dramatically stimulated the expression of PPARα and its target gene CPT1a, and inhibited the expression of ACC (Figure 3E,F). The GLP-1 antagonist Ex-9 reversed the effects of Ex-4 on the expression of ACC and CPT1a.

### PPARα Silencing Inhibits the Effects of GLP-1 on Lipid Metabolism, Oxidative Stress and Hepatic Cell Apoptosis *In Vitro*

**FIGURE 4:**
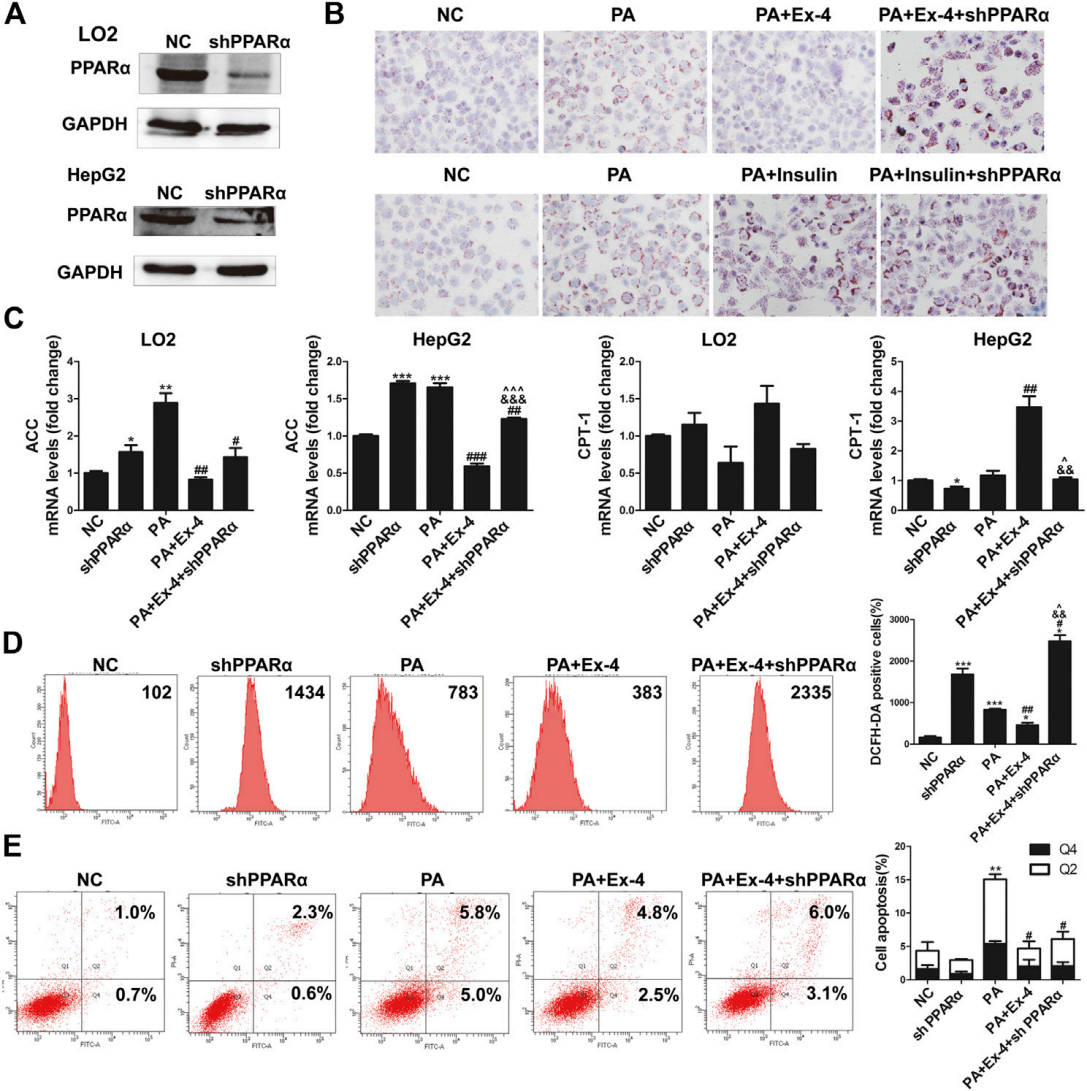
Silencing PPARα aggravates PA increased of oxidative stress and hepatic cell apoptosis. **(A)** Western blot indicates that shPPARα reduced the expression of PPARα in hepatic cells. **(B)** Oil Red O staining shows that shPPARα reversed the Ex-4 alleviated lipid accumulation in hepatic cells. **(C)** Real-time PCR shows that shPPARα reversed the effects of Ex-4 on the expression of ACC and CPT-1 (^*^ *p* < 0.05, ^**^ *p* < 0.01, ^***^ *p* < 0.001 vs. NC group; ^#^ *p* < 0.05, ^##^ *p* < 0.01, ^###^ *p* < 0.001 vs. PA group; ^&^ *p* < 0.05, ^&&^ *p* < 0.01, ^&&&^ *p* < 0.001 vs. PA + Ex-4 group; ^^^ *p* < 0.05, ^^^^ *p* < 0.01, ^^^^^ *p* < 0.001 vs. shPPARα group). **(D)** Flow cytometry shows DCFH-DA positive cells indicate that shPPARα reversed the Ex-4 suppressed ROS level in indicated hepatic cells. Bars on the right panel represent the DCFH-DA positive cells (^*^ *p* < 0.05, ^**^ *p* < 0.01, ^***^ *p* < 0.001 vs. NC group; ^#^ *p* < 0.05, ^##^ *p* < 0.01, ^###^ *p* < 0.001 vs. PA group; ^&^ *p* < 0.05, ^&&^ *p* < 0.01, ^&&&^ *p* < 0.001 vs. PA + Ex-4 group; ^^^ *p* < 0.05, ^^^^ *p* < 0.01, ^^^^^ *p* < 0.001 vs. shPPARα group). **(E)** Annexin V-FITC/PI flow cytometry assay showed the effects of shPPARα on Ex-4 suppressed apoptosis. Bars on the right panel represent percentage of cells in Q2+Q4 (^*^ *p* < 0.05, ^**^ *p* < 0.01, ^***^ *p* < 0.001 vs. NC group; ^#^ *p* < 0.05, ^##^ *p* < 0.01, ^###^ *p* < 0.001 vs. PA group).

To further confirm the mediating role of PPARα in GLP-1 stimulated hepatic lipid metabolism, we silenced PPARα using short hairpin (sh)PPARα in LO2 and HepG2 cells. Administration of shPPARα dramatically inhibited the expression of PPARα protein in both cell lines (Figure 4A). Silencing of PPARα removed the inhibitory effect of GLP-1 on lipid accumulation in hepatic cells (Figure 4B, upper panel). Silencing of PPARα also aggravated insulin stimulated lipid accumulation in hepatic cells (Figure 4B, lower panel). Moreover, the basal expression of ACC was increased and CPT1a was decreased in the two hepatic cell lines when PPARα was silenced. Knockdown of PPARα suppressed the Ex-4 stimulated expression of CPT1a, while stimulation of Ex-4 inhibited the expression of ACC in hepatic cells (Figure 4C).

As detected by the ROS assay, silencing of PPARα reversed the Ex-4 suppressed oxidative stress, and the number of DCFH-DA-positive cells in the shPPARα group was even larger than that in the PA group (Figure 4D). Flow cytometry revealed that silencing of PPARα reversed the Ex-4 rescued apoptosis of hepatic cells (Figure 4E).

### GLP-1R-Dependent AMPK Signaling Pathway Is Involved in GLP-1 Regulated Lipid Metabolism and Liver Injury

**FIGURE 5:**
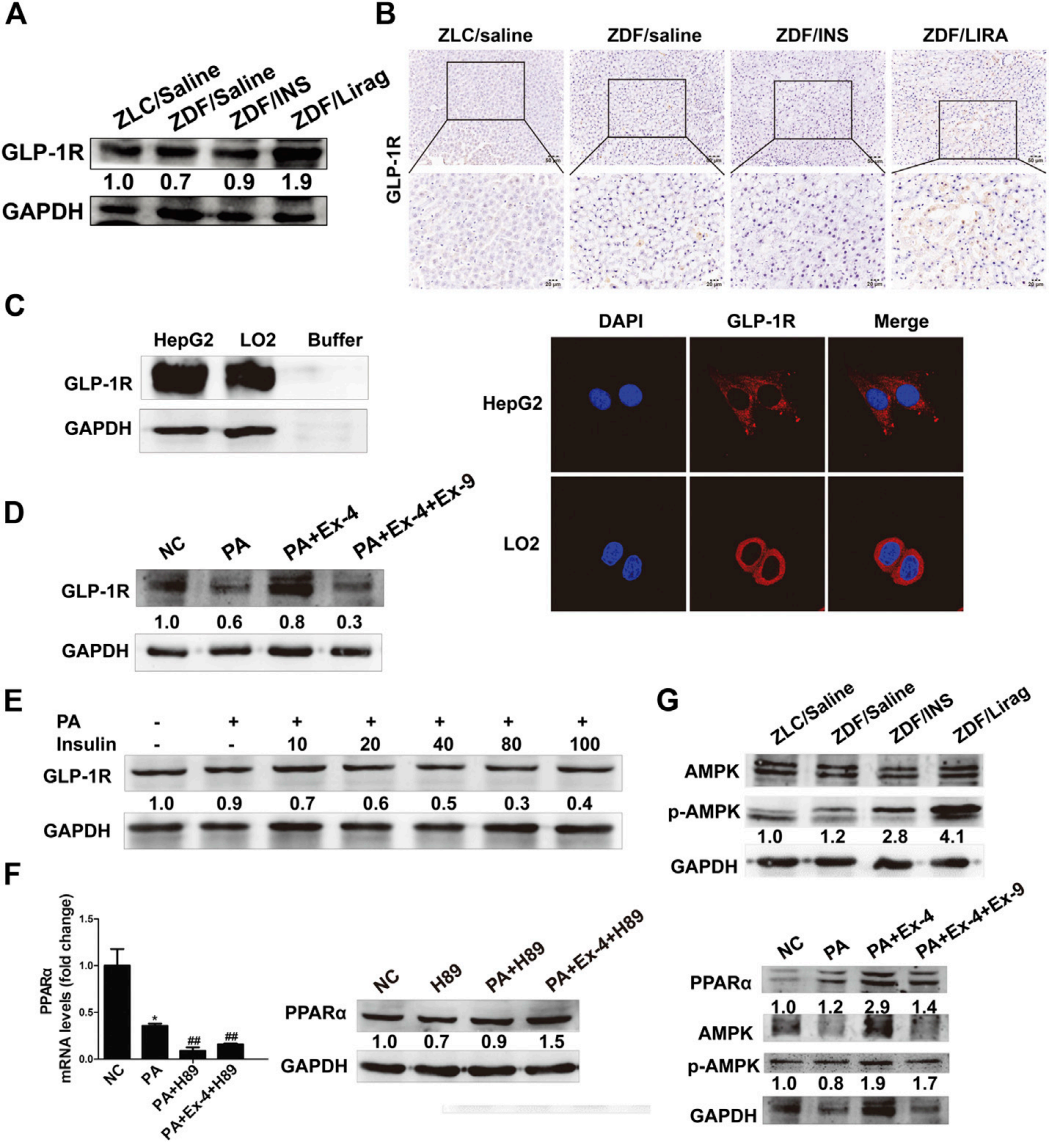
GLP-1 regulates lipid metabolism and liver injury via a GLP-1R-dependent AMPK signaling pathway. **(A)** Western blot shows the expression of GLP-1R in the hepatic tissues of lean and indicated ZDF rats. **(B)** IHC staining shows the expression of GLP-1R in the hepatic tissues of lean and indicated ZDF rats. **(C)** Western blot and immunofluorescence staining shows the expression of GLP-1R in hepatic cell lines. **(D)** The expression of GLP-1 in Ex-4 and Ex-9 treated hepatic cells. **(E)** The expression of GLP-1R after insulin treatment. **(F)** Real-time PCR and western blot assay shows the expression of PPARα after the treatment of H89 (^*^ *p* < 0.05, ^**^ *p* < 0.01, ^***^ *p* < 0.001 vs. NC group; ^#^ *p* < 0.05, ^##^ *p* < 0.01, ^###^ *p* < 0.001 vs. PA group). **(G)** Western blot assay shows the activation of AMPK signaling in lean rats, indicated ZDF rats and hepatic cells.

GLP-1 receptor expression was confirmed in both hepatic tissues and cells. Among the ZDF rats, the liraglutide group showed the highest expression of GLP-1R (Figure 5A). IHC staining of GLP-1R showed the same trends of expression in lean rats and ZDF rats with different treatments (Figure 5B). High GLP-1R expression was also detected in LO2 and HepG2 cells detected by western blot and immunofluorescence staining (Figure 5C). Ex-4 dramatically stimulated the expression of GLP-1R in hepatic cells, while Ex-9 totally abolished the Ex-4 induced GLP-1R increase (Figure 5D). The PA diet suppressed the expression of GLP-1R. Interestingly, insulin significantly inhibited the expression of GLP-1R in a dose-dependent manner (Figure 5E). H89, an inhibitor of protein kinase A, blocked the basal and Ex-4 stimulated expression of PPARα (Figure 5F). The AMPK pathway is involved in GLP-1R mediated downstream signaling both *in vivo* and *in vitro*. AMPK signaling was active in the hepatic tissues of liraglutide-treated ZDF rats compared with lean rats and other antidiabetic drug treated ZDF rats (Figure 5G, upper panel). Consistent with this, Ex-9 completely blocked the Ex-4 activated AMPK signaling (Figure 5G, lower panel).

## Discussion

NAFLD is a common liver disease worldwide, ranging from NAFL to NASH. Fatty liver may not severely disturb liver functions. However, steatohepatitis combined with inflammation and fibrosis can progress to liver damage with many complications (Friedman et al., 2018). Accumulating evidence indicates that T2DM is closely associated with NAFLD. T2DM has been identified in 23 and 47% of patients with NAFL and NASH, respectively (Younossi et al., 2016). No effective pharmacotherapy has been approved for NAFLD, let alone T2DM with NAFLD. The use of GLP-1 agonist as a novel antidiabetic drug can alleviate lipid accumulation and inflammation in hepatocytes (Cuthbertson et al., 2012; Wang et al., 2014; Xu et al., 2014). However, the underlying mechanisms are not fully understood.

Directly modified from native GLP-1, liraglutide shares 97% homology with native GLP-1, and could successfully protract the GLP-1 activity, resist DPP4 activity and limit renal clearance (Aroda, 2018). In our previous study, besides reducing blood glucose, the protective role of liraglutide in different organs, including the bone, heart, kidney and liver were studied (Yang et al., 2020). Besides glycaemic control, histological studies indicated that liraglutide could also dramatically alleviate fatty liver in ZDF rats. To our surprise, ZDF rats in the insulin group, which acted as a control to remove the influence of blood glucose, displayed severe hepatic steatosis without significantly influencing food intake compared with the control ZDF rats. As the combination treatment tends to be the most promising solution suggested by the latest AACE/ACE clinical practice guidelines, we proposed that the combined liraglutide and insulin treatment might alleviate insulin aggravated hepatic steatosis. As expected, our results indicated that liraglutide could dramatically alleviate the insulin aggravated fatty liver *in vitro*. Although the role of liraglutide in alleviating hepatic fatty accumulation has been controversial, the idea is consistent with a recent reports that described that liraglutide combined with metformin significantly reduced body weight and intrahepatic lipid and visceral adipose tissue in patients with T2DM and NAFLD (Tang et al., 2015; Yan et al., 2018). Despite the fact that insulin reduces total liver fat in a previous clinical trial, it is still reasonable to believe that insulin, as a recognized anabolism stimulator, is associated with weight gain and could increase hepatic fatty synthesis by activating lipogenic and glycolytic enzymes (Tilg and Moschen, 2008; Tang et al., 2015; Smits et al., 2016).

Clinically, liraglutide could reduce body weight and hepatic fatty accumulation (Kelly et al., 2020). However, researches on the underlying mechanisms are still limited. Liraglutide treatment could improve insulin sensitivity, accompanied with the reduced expression of the phosphorylated Acetyl-CoA carboxylase-2 and upregulation of long chain acyl CoA dehydrogenase (LCAD) (Zhou et al., 2019). The administration of liraglutide could activate the expression and downstream signaling of GLP-1R (Kushima et al., 2017; Cheng et al., 2019). GLP-1 receptor belongs to a class B G-protein-coupled receptor family that signals primarily through the stimulatory G protein Gs, and activates the protein kinase A (PKA), extracellular signal-regulated kinase (ERK)1/2 and phosphoinositol 3 kinase (PI3K)/protein kinase B (PKB) (Thorens, 1992; Arnette et al., 2003; Briaud et al., 2003; Zhang et al., 2017). The expression of GLP-1R have been demonstrated in many tissues and organs, such as adipocyte, hepatocyte and endothelial cells (Kushima et al., 2017). Consistently, we confirmed the expression of GLP-1R and the GLP-1 (liraglutide or Ex-4) upregulated expression of GLP-1R in hepatic tissues and cells. Meanwhile, PKA signaling directly downstream of GLP-1R is also activated, indicating a direct involvement of hepatic GLP-1R in GLP-1 mediated alleviation of fatty liver and liver injury. In this study, we also demonstrated the inhibition of hepatic GLP-1R by insulin for the first time. Thus, insulin might stimulate hepatic fatty deposition and liver injury by blocking GLP-1R mediated hepatic lipolysis. The heterotrimeric protein AMPK, consisting of a catalytic α and regulatory β and γ subunits, is ubiquitously expressed and plays a pivotal role as a regulator of energy homeostasis, such as mitochondrial biogenesis, fatty acid synthesis and glucose uptake (Hardie et al., 2012). Liraglutide could activate the AMPK during glucose transportation (Andreozzi et al., 2016). AMPK signaling is also involved in liraglutide stimulated fatty degradation in both hepatic and adipose tissues (He et al., 2016; He et al., 2020). DPP4 inhibitor, which could inhibit the degradation of endogenous GLP-1, can stimulate the activation of AMPK and fatty degeneration in adipocyte (Cheng et al., 2019). In our current study, we confirmed the involvement of AMPK signaling in liraglutide stimulated fatty degradation.

The balance between fatty synthesis and lipolysis, which is catalyzed by related enzymes, determines fatty accumulation in the liver. PPARα is a ligand-activated transcription factor that along with PPARδ and PPARγ belongs to the NRIC nuclear receptor subfamily (Pawlak et al., 2015). PPARα can activate lipid metabolism by stimulating the metabolism of fat by peroxisomal and mitochondrial fatty acid β-oxidation and microsomal ω-oxidation (Misra and Reddy, 2014; Kersten and Stienstra, 2017). PPARs can also activate the metabolism of carbohydrates and lipids by regulating its target genes, including UCP2, HSL, CPT1 and ACC (Li et al., 2019). Presently, PPARα was overexpressed in the liver of liraglutide- and saxagliptin-treated ZDF rats. Oil Red O staining demonstrated that shPPARα could relieve the PA induced hepatic fatty deposition, indicating a role in mediating GLP-1 mediated remission of fatty liver. Moreover, Ex-4 stimulated the expression of PPARα and its downstream CPT1a, while inhibiting the expression of its downstream ACC. Administration of the GLP-1 antagonist Ex-9 abolished the effects of Ex-4 on PPARα, CPT1a and ACC. Consistent with previous studies (Ip et al., 2003; Gervois et al., 2004), our findings strengthened the mediatory role of PPARα in liraglutide ameliorated fatty accumulation, and confirm the potential of PPARα as a drug target in the management of NAFLD.

Liver injury manifested by hepatic cell apoptosis was also observed in ZDF rats and PA treated liver cells. Liraglutide remarkably reduced apoptosis in hepatic tissues of ZDF rats. Oxidative stress contributes to liver injury. The GLP-1 agonist Ex-4 dramatically reduced the ROS level in PA treated hepatic cells, whereas insulin increased the basal ROS level. This is because insulin increased hepatic steatosis could also elevate the ROS level. TUNEL and flow cytometry analyses demonstrated an inhibitory role of liraglutide in liver apoptosis *in vivo* and *in vitro*. Although insulin did not stimulate basal hepatic cell apoptosis, it aggravated PA induced liver injury. Blockage of GLP-1R by Ex-9 dramatically restored the Ex-4 reduced ROS levels and liver cell apoptosis, indicating that GLP-1 reduced liver cell apoptosis by decreasing ROS levels. Moreover, we also revealed the mediatory role of PPARα in alleviating liver injury.

**FIGURE 6:**
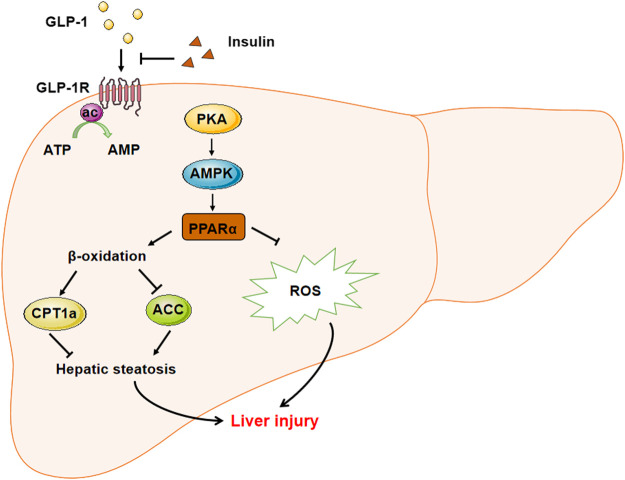
The sketch map demonstrated the mechanism involved in liraglutide alleviated hepatic steatosis and liver injury. GLP-1 agonist liraglutide could directly act on hepatic GLP-1R to stimulate the expression of PPARα through activating the PKA-AMPK signaling pathway. PPARα could stimulate the β-oxidation to relieve hepatic steatosis through stimulating the expression of CPT1a and decreasing that of ACC. Meanwhile, PPARα could also decrease the intracelluar ROS level to alleviate the liver injury. On the other hand, insulin could suppress the expression of GLP-1R to inhibit its downstream signaling, while the administration of GLP-1 could recover the insulin aggravated hepatic steatosis.

In summary, this study is the first exploration of the liraglutide and insulin combination treatment to alleviate the basal insulin aggravated NAFLD in T2DM. The underlying mechanism was also clarified (Figure 6). PPARα mediated the GLP-1-regulated fatty metabolism as a transcription factor, and alleviated liver injury by reducing ROS-related apoptosis. Our findings also provide the first evidence of the inhibitory role of insulin on the basal expression of GLP-1R, which might explain why liraglutide could alleviate the insulin aggravated hepatic steatosis. The data provide a theoretical basis for the clinical use of liraglutide combination to treat T2DM.

## Data Availability

The original contribution presented in the study are included in the article/Supplementary Material, further inquries can be directed to the corresponding authors.
